# The revised short-form of the Eating Beliefs Questionnaire: Measuring positive, negative, and permissive beliefs about binge eating

**DOI:** 10.1186/s40337-018-0224-0

**Published:** 2018-11-06

**Authors:** Amy L. Burton, Maree J. Abbott

**Affiliations:** Clinical Psychology Unit, School of Psychology, The University of Sydney, 94 Mallett St, Camperdown, 2050 Australia

**Keywords:** Binge eating, Beliefs, Self-report, Questionnaire, Factor analysis, Validity

## Abstract

**Background:**

The Eating Beliefs Questionnaire (EBQ) is a self-report assessment tool that measures *positive* and *negative* beliefs about food and eating that are believed to play a key role in maintaining binge eating behaviour that occurs in individuals with Bulimia Nervosa, Binge Eating Disorder and other atypical eating disorders. The present study aimed to further refine this measure with the addition of a third scale to assess *permissive* beliefs about eating, also thought to play a crucial role in the maintenance of binge eating. Permissive beliefs are defined as beliefs about eating that provide justification for the individual to engage in a binge eating episode.

**Methods:**

After consultation with the literature and endorsement from 10 experts in eating disorders, 19 permissive belief items were generated. Eight hundred eighty-three participants were recruited to complete a test battery online that included the EBQ and the new permissive items.

**Results:**

An exploratory factor analysis (*n* = 441) found a three-factor solution (positive, negative and permissive beliefs) explaining 63.4% of variance. A confirmatory factor analysis (*n* = 442) provided support for the three-factor model, with the data best supporting a shorter 18-item questionnaire. The revised scale demonstrated good internal consistency, as well as good convergent validity with measures of related eating disorder symptoms, emotional regulation, mood and anxiety.

**Conclusions:**

With the addition of a third scale to measure permissive beliefs, the revised short-form of the EBQ offers clinicians and researchers a brief comprehensive tool for the measurement of positive, negative and permissive beliefs about binge eating.

**Electronic supplementary material:**

The online version of this article (10.1186/s40337-018-0224-0) contains supplementary material, which is available to authorized users.

## Plain English summary

In this paper we extend upon an existing self-report questionnaire that assesses beliefs about eating that are thought to maintain binge eating behaviour in eating disordered individuals; The Eating Beliefs Questionnaire (EBQ). The revised questionnaire consists of three scales each measuring a different type of belief about eating: positive beliefs about binge eating (beliefs related to eating helping to reduce unpleasant emotions), negative beliefs about binge eating (beliefs related to not being able to control one’s eating), and permissive beliefs about binge eating (beliefs related to allowing oneself to commence or continue a binge episode). We assess the psychometric properties of the new three subscale EBQ; the results indicate that the measure is valid and reliable. The three subscale EBQ provides a tool for clinicians and researchers to measure the presence of positive, negative and permissive beliefs about binge eating which can be used to help guide treatment or measure the shift in these beliefs over the course of treatment.

## Background

Binge eating is a symptom of disordered eating present in individuals with bulimia nervosa (BN), binge eating disorder (BED), anorexia nervosa binge/purge type (AN-BP) and atypical eating disorders [1]. Binge eating also occurs in the general community at a sub-clinical level [2]. Results from a series of sequential community studies have demonstrated that the reported rates of binge eating have significantly increased over time, with more recent prevalence estimates indicating that between 4.9 to 7.2% of Australians engage in binge eating episodes [3, 4]. The impact of binge eating on the individual, as well as broader society, is substantial, with numerous serious psychological and physical health complications associated with binge eating [5–10]. In addition, recurrent binge eating is associated with poorer quality of life and impaired social functioning [3, 11].

Given the high rates of binge eating and the significant impact that this behaviour has on affected individuals, it is clear that effective targeted interventions are required. Binge eating is notoriously difficult to treat; even when the best available evidence-based treatments for BN and BED are implemented (e.g., Enhanced CBT [12, 13]), research has demonstrated that only between 40 and 50% of participants have ceased binge eating at follow-up [13–17]. In order to develop more effective treatments, it is important to first have a sound understanding of the maintaining factors that underlie binge eating behaviour. However, at present this remains an area of uncertainty in the literature. While most influential cognitive models of binge eating focus on the role of restrictive eating behaviour, low self-esteem, and overvaluation of body shape and weight, few also consider the core cognitive processes that function to maintain binge eating (see Burton & Abbott [18] for a review). One exception that emphasises the role of specific cognitions in maintaining binge eating behaviours is Cooper, Wells and Todd’s cognitive model of BN [19]. In this model, the cycle of bulimic behaviours (bingeing and purging) are driven by negative core beliefs, beliefs about eating/food and a series of metacognitive beliefs (beliefs about the symptoms of binge eating and processes). Specifically, metacognition is conceptualised as consisting of thoughts and beliefs related to the monitoring, control, interpretation and appraisal of cognitive events and behaviours [20]. Cooper, Wells and Todd [19] identify three main types of metacognitive beliefs that act together to maintain binge eating – positive, negative and permissive beliefs about food and eating. According to this model, a binge eating episode is triggered by a distressing event which activates a negative belief about the self as an acceptable person, such as ‘I’m unlovable’ or ‘I’m a failure’. The activation of these negative self-beliefs is accompanied by feelings of anxiety, depression or guilt. The model proposes that affected individuals commence binge eating as a means to cope with these unpleasant emotions, and that the process of binge eating reduces the intensity of the emotional states in the short term, which further reinforces positive beliefs about eating. Positive beliefs relate to the perceived benefits of binge eating, particularly its perceived role in reducing emotional distress (e.g., ‘eating helps me to cope with negative feelings’). Cooper et al. describe a conflict experienced by those with BN between positive and aversive beliefs about eating, such as “eating will help me to cope, but, eating will also make me fat”. This conflict is thought to cause additional distress. As a result, *permissive beliefs* and *negative (no control) beliefs* about eating develop as a means of attempting to reduce this distress. Permissive beliefs are those which allow/permit the individual to commence or continue a binge eating episode, but do not address beliefs about one’s ability to control urges to binge (e.g., ‘I deserve to have a pleasure like bingeing’). Negative (no control) beliefs relate to the perceived inability of the individual to control themselves in relation to food and eating in terms of resisting urges to eat and/or stopping eating once an episode of binge eating has started (e.g., ‘Once I start eating I can’t stop’). The activation of permissive beliefs and negative beliefs trigger binge episode by means of the individual ‘permitting’ themselves to commence a binge and/or feeling unable to stop themselves from commencing a binge. In turn, the act of binge eating then further activates negative self-beliefs (e.g., ‘I am weak’), and aversive thoughts about eating (e.g., ‘I’ll get fat’), which either leads to further binge episodes or, for those with BN, compensatory behaviours such as purging. The cycle is further reinforced as the behaviours of bingeing and purging activates and/or further strengthens the individual’s negative self-beliefs and beliefs of no control over eating (negative beliefs). Therefore, Cooper et al. suggest that it is the combination and interaction of core beliefs (negative self-beliefs) and the three types of metacognitive beliefs (positive, permissive and negative beliefs about eating) that are proposed to maintain binge eating behaviour.

There are two existing measures that assess the metacognitive beliefs about food, disinhibited eating and binge eating that are hypothesised in the cognitive model of BN [19] to play a key role in maintaining binge eating behaviour [21, 22]; the eating disorder thoughts questionnaire (EDTQ) [23] and the Eating Beliefs Questionnaire (EBQ) [22]. These two measures bear similarities but measure different constructs. The EDTQ is a 26-item self-report questionnaire that measures thoughts about the negative/aversive consequences of eating (e.g., “I’ll get fat”), the positive consequences of eating (e.g., “If I eat it will stop the pain”), and permissive thoughts (e.g., “One more bite won’t hurt”). In contrast, the EBQ is a 16-item self-report questionnaire that measures beliefs about no control over eating (e.g., “Once I start eating I can’t stop”) and positive beliefs about eating (e.g., “Eating helps me cope”). While both measures assess ‘positive beliefs’, the EDTQ positive thoughts scale includes items that do not match the original definition of positive thoughts (i.e., thoughts that relate to the perceived benefits of eating), for example “go on, eat more to punish yourself” and “its not me doing this” [23], whereas all items in the EBQ positive beliefs scale relate directly to perceived benefits of eating, especially with regard to the role of eating in regulating emotion [24]. At present, neither the EDTQ or the EBQ provide a valid and reliable measurement of the *three* types of metacognitions that maintain binge eating, that is, positive beliefs about the perceived benefits of eating (‘positive beliefs’), beliefs about having no control over eating (‘negative (no control)’) and beliefs that make it easy to eat or keep eating (‘permissive beliefs’).

The EBQ has been found to be a valid and reliable measure of positive and negative (no control) beliefs about eating in both clinical and non-clinical samples with demonstrated evidence of excellent internal consistency (α = .94), good test-retest reliability (*r* = .91) and sensitivity to eating disorder treatment [22]. In addition, EBQ scores were found to significantly and positively correlate with binge eating episode frequency, increases in body mass index (BMI), and measures of eating disorder behaviours and related psychopathology, as well as measures of depression, anxiety and stress [22]. While the EBQ provides a valid and reliable measure of positive and negative (no control) beliefs about eating, it does not provide a measure of the third type of metacognitive belief described in Cooper et al.’s model which is also implicated in the maintenance of binge eating, that is, *permissive* beliefs about eating. Given that Cooper et al.’s model [19] emphasises the role of all three types of metacognitive beliefs in the maintenance of binge eating behaviour, we believe that extending the EBQ to also include a measure of permissive beliefs would offer a more thorough assessment of the cognitions hypothesised to maintain binge eating behaviour, thereby providing one questionnaire that can reliably measure all three types of maintaining beliefs. We hope that by producing a more comprehensive questionnaire for the assessment of metacognitive beliefs about binge eating, the EBQ will provide a valuable tool for clinicians using cognitive therapy to treat binge eating, and for researchers investigating the cognitive underpinnings of eating disorder psychopathology and related processes.

### Aims and hypotheses

The first aim of this study was to develop a third subscale for a revised version of the EBQ that aimed to measure permissive beliefs about eating/bingeing as defined by Cooper et al. [19]. This study also aimed to investigate the factor structure of the revised EBQ and examine its psychometric properties. Finally, this study aimed to present a short-form of the three-factor EBQ. It is predicted that the inclusion of the additional items will result in a three factor solution: positive, negative and permissive beliefs about eating. It is also predicted that the revised version of the EBQ will demonstrate good psychometric properties including adequate internal consistency, and convergent validity. More specifically, we hypothesised that the revised EBQ will demonstrate positive correlations with measures of constructs relevant to binge eating including negative affect, eating disorder symptoms and behaviours, eating disorder related cognitions, negative core beliefs, and poor emotional regulation [18]. Based on the findings of previous research [23], we predicted that the permissive belief scale will not correlate with body mass index (BMI) or with a measure of dietary restraint, but will correlate with the other included measures of eating disorder symptoms, behaviours, cognitions and related constructs.

### Study 1: Item development and content validity

#### Development of new items

Originally, the authors generated 20 items based on the literature on permissive beliefs. The authors contacted ten mental health professionals (clinical psychologists and psychiatrists) who have training and experience in the assessment and psychological treatment of eating disorders to ask if they would provide feedback on the proposed items, all agreed to participate. These ten expert mental health professionals all either work predominantly with people with eating disorders or have a specific research interest in eating disorders. Three additional items were suggested by the experts and ten items were re-worded following feedback from the experts.

#### Assessment of content validity

The ten experts were then asked to provide their assessment of the content validity of the 23 revised items by providing a rating of ‘relevance’ to permissive beliefs for each of the items. Experts were provided with a definition of permissive beliefs to refer to when making their assessment of each item’s relevance: “Permissive beliefs - beliefs which allow individuals to “permit” themselves to commence or continue a binge eating episode, but do not address beliefs about one’s ability to control urges to binge. For example, permissive beliefs might relate to allowing oneself to binge after a stressful or difficult experience or as a means of having something positive in one’s life”. Experts rated each item on a 3-point likert scale (1 = not relevant, 2 = somewhat relevant, 3 = very relevant) for their relevance to permissive beliefs. Four items were deemed to be *not relevant* by at least 30% of the experts, and these items were removed. Nineteen items were rated as being relevant to permissive beliefs by at least 8/10 of the experts.

In total there were 19 new items developed by the authors, with the help of experts in eating disorders, selected and added to the existing 16 EBQ items with the aim of providing an additional subscale to assess permissive beliefs about binge eating. This created a three scale EBQ assessing positive, negative and permissive beliefs consisting of 35 items. It was necessary to first examine the factor structure and psychometric properties of this three scale EBQ.

### Study 2: Factor structure, internal consistency and scale validity

#### Participants

In total, 907 participants took part in the study (72% female, mean age = 20.38 years, SD = 4.88 years). For the purposes of our data analysis, we required complete data sets with no missing data. Therefore the incomplete data from 24 participants were removed from further analyses, leaving the complete data from a total of 883 participants which were included in this study (demographic information summarised in Table 1). Participants were recruited from a sample of first year psychology students at the University of Sydney, who participated in exchange for course credit, and from the general community by means of an advertisement placed on Australian online community pages. Informed consent was obtained from all individual participants included in the study.

**Table 1:** Demographic Information for Participant Samples Included in Analyses

Sample	*n*	Mean age in years (SD)	Mean BMI (SD)	% Female	Mean OBEs in past 28 days (SD)
Total sample	883	20.38 (4.91)	22.21 (3.82)	72.0	2.57 (5.74)
University students	767	19.37 (3.46)	21.99 (3.52)	71.2	2.48 (5.21)
General community	116	27.11 (7.27)	23.68 (5.15)	77.6	3.17 (8.44)
BE	169	20.28 (5.03)	23.29 (4.58)	91.0	10.05 (6.31)
Non-BE	481	20.63 (5.30)	21.78 (3.40)	65.9	0.00

##### Sub-samples based on self-reported BE status

Two sub-samples of differing self-reported binge eating status were identified using the participants’ responses on the Eating Disorder Examination Questionnaire (EDE-Q; refer to Materials for more information); non-binge eating (Non-BE) and binge eating (BE) sample. Non-BE sub-sample: Of the total sample, 481 participants (54.47%) reported that they had engaged in 0 episodes of objective binge eating (OBEs; objective overeating with a sense of loss of control) over the previous 28 days. BE sub-sample: Of the total sample, 169 participants (19.13%) reported that they had engaged in ≥ 4 OBEs over the previous 28 days. Demographic information of these sub-samples are summarised in Table 1.

#### Procedure

Participants completed a battery of questionnaires online using Qualtrics Software and they measured/recorded their weight and height to determine their BMI. Participants from the university sample (*n* = 767) completed a full-test battery consisting of all the measures described. Participants from the community sample (*n* = 116) completed a brief test-battery that consisted of only demographics items, the EBQ items, the Depression Anxiety Stress Scale (DASS-21) and the Eating Disorders Examination Questionnaire (EDE-Q). To reduce potential fatigue effects the order of presentation of the questionnaires was randomised and participants could take breaks as required.

#### Materials

To provide a thorough assessment of the EBQ’s construct validity, the test battery included a range of measures which assess known correlates of binge eating and/or eating disorders [18], including body mass (BMI), overall mood and distress (DASS-21), eating disorder symptoms and related behaviours (EDE-Q and Dutch Eating Behaviour Questionnaire), eating disorder related cognitions (EDE-Q, Eating Disorders Thoughts Questionnaire), negative core-beliefs (Eating Disorders Core Beliefs Questionnaire), and poor distress tolerance (Difficulty with Emotional Regulation Scale).

##### *The Eating Beliefs Questionnaire (EBQ)* [21, 22]

The EBQ is a 16-item self-report measure that assesses positive and negative metacognitions about eating and urges to eat when not hungry. Participants are asked to rate how much they agree with each of the items from 1 (strongly disagree) to 5 (strongly agree). With the addition of the 19 permissive items (see Study 1 for a description of the development of these items), the EBQ utilised in this study consisted of 35 items.

##### Body mass index (BMI)

Participants recorded their height and weight so that their BMI could be calculated using the formula: BMI = kg/m^2^. BMI categories are used as an indication of whether an individual’s body mass is within healthy limits. Having a BMI higher or lower than the healthy/normal range increases the risk of illness including diabetes and cardio-vascular disease [25].

##### *The Eating Disorder Examination Questionnaire (EDE-Q, version 6.0)* [26]

The EDE-Q is the self-report questionnaire derived from the Eating Disorder Examination interview (EDE) [27]. The EDE**-**Q assesses frequency and severity of eating disorder symptoms (including binge eating) that the respondent has experienced over the previous 28 days. Participants rate each item with a score between 0 and 6, where high scores indicate a greater frequency or severity of symptoms (e.g., fasting, purging, driven exercise and binge eating). Item 14 of the EDE-Q 6.0 assesses frequency of objective binge eating episodes (OBEs; objective overeating with a sense of loss of control), responses on this item were used to create a subgroup of participants who self-reported to have engaged in at least four OBEs over a 28-day period (the frequency of four OBEs was chosen as this is the minimum frequency of OBEs to meet DSM-5 criteria for BN or BED) [1]. Also, the EDE-Q provides a global ED severity score as well as four subscale scores that include dietary restraint, eating concern, weight concern and shape concern. Various studies have found the EDE-Q to be a valid and reliable self-report measure [24]. Cronbach’s alpha of .94 for the EDE-Q global score in the current study.

##### *Depression Anxiety Stress Scale (DASS-21)* [28]

The DASS-21 is a self-report measure that assesses the presence of depression, anxiety and stress symptoms occuring over the past fortnight. The DASS-21 is a valid and reliable measure with good psychometric properties [29]. Cronbach’s alpha of .93 for the total score in the present study.

##### *Dutch Eating Behaviour Questionnaire (DEBQ)* [30]

The DEBQ is a self-report measure that assesses eating behaviours and attitudes towards eating. The present study utilised two of the three DEBQ scales: the emotional eating scale that consists of 13 items measuring the impact of emotional cues on eating behaviour, and also the external eating scale that consists of 10 items measuring the impact of external/environmental cues on eating behaviour. Cronbach’s alpha of .95 for the emotional eating subscale and Cronbach’s alpha of .83 for the external eating subscale in the present study.

##### *Eating Disorders Core Beliefs Questionnaire (ED-CBQ)* [31]

The ED-CBQ provides an assessment of the presence of core beliefs about the self that are thought to be relevant to eating disorders. The ED-CBQ is a 40-item measure consisting of 5 subscales, each assessing a sub-type of relevant core beliefs (self-loathing, unassertiveness/inhibited, high standards for self, demanding/need help and support, and abandoned/isolated). Participants are asked to provide a rating of how often they believe/feel each of the core beliefs presented to be true of them. The ED-CBQ has been found to demonstrate adequate internal consistency and contruct validity [31]. Cronbach’s alpha was .92 for the total score in the present study.

##### *Eating Disorder Thoughts Questionnaire (EDTQ)* [23]

The EDTQ is a 26-item self-report measures that assesses the presence of certain beliefs held by individuals with eating disorders. Types of beliefs assessed include beliefs about the positive and negative consequences of eating, and permissive thoughts that allow individual to commence or continue eating. The EDTQ was found to demonstrate good internal consistency, and good construct validity, criterion validity and discriminant validity in the initial validation study [23]. Cronbach’s alpha was .96 for the total score in the present study.

##### *Difficulty in Emotion Regulation Scale (DERS)* [32]

The DERS provides a tool for the measurement of difficulties in emotion regulation. This 36-item self-report measure consists of 6 subscales assessing different types of difficulties with emotion regulation (non-acceptance of emotional responses, difficulty engaging in goal-directed behaviour, impulse control difficulties, lack of emotional awareness, limited access to emotional regulation strategies, and lack of emotional clarity). Participants provide a rating on a 5-point scale of how frequently they experience the behaviour described in each item. Higher scores indicate poor emotion regulation. The DERS subscales have been found to demonstrate good internal consistency, test–retest reliability, and adequate construct and predictive validity [32]. Cronbach’s alpha of .90 for the whole measure in the present study.

#### Analyses

##### Factor analyses

The data were cleaned prior to pooling, any participants with missing data were removed from further analysis, and the distribution was inspected for normality. In total, the sample consisted of data from 883 participants (767 university students and 116 community participants). Half of this data was used for the Exploratory Factor Analysis (EFA) and the other half used for the Confirmatory Factor Analysis (CFA). The EFA analyses are based on *n* = 441 (116 community, 325 university students), and the CFA analyses are based on *n* = 442 university student participants. This provided two samples for factor analysis of adequate size with 12–17 participants per item for each analysis [33–36]. It was important that the sample used in the CFA came from the same population pool (in this case, the first year university student population) in order to provide a homogenous sample [34, 37]. Whether the data from a university student participant was used for the EFA or the CFA was randomised. The SPSS v22 program was used to conduct an EFA to examine the factor structure of the revised EBQ. The model was built using a Maximum Likelihood method and Promax rotation. For the EFA, the criteria for retaining items that were applied was standardized regression weights of >.40 and communality >.40, in addition to this items that equally loaded on to two factors were not retained. The AMOS v12 program [38] was used to conduct the CFA on the EBQ items to evaluate the fit of the data to the hypothesised three factor model, with higher-order factor of ‘Eating Beliefs’. The model was built using a Maximum Likelihood method. Model fit was assessed using common goodness-of-fit indices; normed Chi-square (χ^2^/df), the goodness of fit index (GFI), the incremental fit-index (IFI), the Tucker Lewis index (TLo) and the comparative fit index (CFI). A smaller χ^2^/df indicates better fit, with a χ^2^/df of less than 3.00 generally being considered as an indication of good fit [39]. Scores on the GFI, IFI, TLo and CFI range from 0 to 1, with higher scores on these indices indicating better fit, and values over .95 on are reported to indicate good fit [33]. The root mean square error of approximation (RMSEA) and its 90% confidence intervals were also examined to assess fit; RMSEA values of less than 0.06 indicate good fit [33].

##### Psychometric properties

Analyses of the validity of the EBQ were performed using the SPSS v22 program. Internal consistency was tested with Cronbach’s alpha, whereby an alpha between .70 and .95 identifies adequate internal consistency [40]. Criterion, construct and convergent validity were assessed with Pearson correlations. Between group differences were examined using one-way ANOVA and partial eta squared (η_p_^2^) effect sizes are reported, where .01 indicates a small effect, .06 indicates a medium effect and .14 indicates a large effect [41, 42].

## Results

### Exploratory Factor Analysis (EFA)

Examination of the scree-plot indicated a three-factor structure (as hypothesised), so a three-factor EFA was undertaken using Maximum Likelihood and Promax rotation. Ten items with low loadings or cross-loadings were removed from further analysis. A further EFA was conducted on the remaining 25 items. Twelve items related to positive beliefs about disinhibited eating/bingeing loaded on Factor 1, accounting for 44.44% of the total variance explained, 6 items related to permissive beliefs about bingeing loaded onto Factor 2 which explained 11.12% of the total variance, and 7 items relating to beliefs of loss of control over eating (or negative beliefs) accounted for 7.84% of total variance (see bolded items in Table 1). The resultant three-factor structure (positive, permissive and negative beliefs) provided a good fit to the data (χ^2^ = 468.11, df = 207, *p <* .01) and explained 63.40% of the total variance, with all factor loadings and communality (amount of shared variance) above .40, see Table 2.

**Table 2:** Results of an exploratory factor analysis of the EBQ (*n* = 441) standardised regression weights & communality

EBQ items	F1 Positive beliefs	F2 Permissive beliefs	F3 Negative beliefs	Communality (h2)
I’m not able to control my urges to eat	.044	.022	**.701**	.543
Eating helps me to cope	.**746**	.039	.062	.644
My eating will always need to be controlled	−.010	−.207	**.701**	.415
Once I start eating I can’t stop	−.051	.138	**.775**	.653
I have no willpower in relation to food	.055	.035	**.734**	.613
Eating helps to reduce unpleasant physical feelings	**.745**	.082	−.074	.552
Eating means I don’t have to think about negative things	**.741**	−.031	.128	.656
Eating helps to control my emotions	**.852**	−.016	−.012	.703
I can’t control my eating because I am weak	.033	−.120	**.830**	.655
Eating keeps my feelings at a tolerable level	**.710**	−.028	.161	.645
If I don’t control myself I would never stop eating	.038	−.002	**.747**	.591
Eating helps me to cope with negative thoughts	**.894**	−.100	.029	.759
Eating helps me to cope with unpleasant physical sensations	**.728**	.050	.037	.600
There is nothing I can do to stop eating	.000	.249	**.544**	.463
Eating helps me cope with negative feelings	**.922**	−.127	.007	.769
Eating helps to stop feelings that I don’t like	**.867**	−.074	.033	.735
Bingeing is something that I can have for myself	.040	**.704**	.147	.632
I deserve to have a pleasure like binge eating	−.032	**.841**	.045	.715
It’s okay to have the nice experience of binge eating	−.071	**.913**	−.083	.736
Bingeing allows me to have something nice for myself	.009	**.868**	.007	.764
It won’t make a difference if I eat more	.170	**.650**	−.250	.435
I like to binge	−.022	**.771**	.087	.639
Eating stops me from feeling bad	**.797**	.104	−.056	.664
Eating is my best way of coping with unwanted feelings	**.695**	.101	.068	.621
When I eat, things feel better for a while	**.830**	.129	−.165	.650

The bolded data indicates which Factor the item loads on

The three factors of Positive, Permissive and Negative Beliefs were significantly correlated with moderate-to-strong Pearson’s *r*’s: Positive and Permissive, *r =* .44, *p* < .01*;* Positive and Negative, *r =* .59, *p* < .01*;* Negative and Permissive, *r =* .39, *p < .*01. This finding suggests that a higher-order factor structure was plausible (a higher-order factor structure is used when the factors are strongly related and are measuring the same broader construct; in this case the factors are measuring positive, permissive and negative beliefs about eating which are all beliefs about eating, and therefore suggests a possible a higher-order factor of ‘eating beliefs’). This factor structure was examined using confirmatory factor analysis. Furthermore, this three-factor version of the EBQ was very strongly correlated with the original two-factor version of the EBQ, *r* = .96, *p <* .01.

#### Confirmatory factor analysis (CFA)

To assess the goodness of fit of the three-factor solution as determined by the EFA and to confirm that the EBQ provides a single measure of a higher-order factor (Eating Beliefs) with three subfactors that are strongly correlated, a Maximum Likelihood factor analysis was conducted using the AMOS (v12) program. The data from a total of 442 university student participants was used in the CFA analysis as this provided a homogenous sample of adequate size, with 17.7 participants per item on the scale [34, 37, 39]. The results of the CFA found that the three-factor solution as determined by the EFA demonstrated adequate to good fit to the data [33, 37]: χ^2^/df = 2.45, GFI = .89, IFI = .95, TLo = .94, CFI = .95 and the RMSEA = .057 with its 90% CI .052–.063. Refer to Table 3 for the factor loadings, all above .40, and communality, all above .20, for the 25 EBQ items.

**Table 3:** Results of a confirmatory factor analysis of the three-factor 25-item EBQ (*n* = 442) standardised regression weights & communality

EBQ items	F1 Negative beliefs	F2 Positive beliefs	F3 Permissive beliefs	Communality (h2)
I’m not able to control my urges to eat	.729			.532
My eating will always need to be controlled	.462			.214
Once I start eating I can’t stop	.750			.563
I have no willpower in relation to food	.766			.586
I can’t control my eating because I am weak	.779			.606
If I don’t control myself I would never stop eating	.748			.560
There is nothing I can do to stop eating	.699			.488
Eating helps me to cope		.655		.429
Eating helps to reduce unpleasant physical feelings		.688		.474
Eating means I don’t have to think about negative things		.801		.641
Eating helps to control my emotions		.813		.660
Eating keeps my feelings at a tolerable level		.753		.568
Eating helps me to cope with negative thoughts		.866		.750
Eating helps me to cope with unpleasant physical sensations		.735		.540
Eating helps me cope with negative feelings		.848		.719
Eating helps to stop feelings that I don’t like		.851		.725
Eating stops me from feeling bad		.810		.657
Eating is my best way of coping with unwanted feelings		.806		.650
When I eat, things feel better for a while		.761		.579
Bingeing is something that I can have for myself			.733	.537
I deserve to have a pleasure like binge eating			.890	.793
It’s okay to have the nice experience of binge eating			.826	.683
Bingeing allows me to have something nice for myself			.826	.682
It won’t make a difference if I eat more			.463	.214
I like to binge			.671	.450

#### Creating a short-form – The EBQ-18

The authors decided to force evenly sized subscales in order to examine and compare the fit of a short-form version of the measure. Therefore, one item from the Negative Beliefs subscale was deleted, and six items were deleted from the Positive Beliefs subscale. The items chosen for removal from the model either had the lowest factor loadings, the lowest communality (squared multiple correlations) and/or the highest standardised residual scores of the items for that subscale (higher residual indicated a greater amount of error in the model, [43]). Importantly, the psychometric and theoretical value of each of the items was discussed prior to an item being removed from the model. Once these seven items were removed, this left a brief 18-item measure with six items loading on the each of the three factors: Negative, Positive and Permissive Beliefs. A CFA was conducted on this model and the three-factor solution for this brief 18-item version of the measure demonstrated good fit to the data and an improved fit compared to the 25-item version [33, 37]: χ^2^/df = 2.07, GFI = .94, IFI = .97, TLo = .97, CFI = .97, and the RMSEA = .049 with its 90% CI .041–.057. The individual item loadings (standardised regression weight estimates) were significant (.67–.89, *p* < .01), see Table 4, and the loading of the three sub-factors to the higher-order factor were also significant (Negative Beliefs = .81, Positive Beliefs = .83, Permissive Beliefs = .47, all *ps* < .01). Communality for all items was greater than .20, see Table 4. The correlations between the three EBQ subscales were moderate to strong (Positive and Permissive, *r =* .40, *p* < .01*;* Positive and Negative, *r =* .63, *p* < .01*;* Negative and Permissive, *r =* .40, *p < .*01). The final model represents a brief 18-item questionnaire with three factors.

**Table 4:** Results of a confirmatory factor analysis of the EBQ-18 (n = 442) standardised regression weights & communality

EBQ items	F1 Negative beliefs	F2 Positive beliefs	F3 Permissive beliefs	Communality (h2)
I’m not able to control my urges to eat	.728			.531
Once I start eating I can’t stop	.748			.559
I have no willpower in relation to food	.763			.582
I can’t control my eating because I am weak	.780			.609
If I don’t control myself I would never stop eating	.745			.555
There is nothing I can do to stop eating	.705			.497
Eating means I don’t have to think about negative things		.806		.649
Eating helps to control my emotions		.830		.689
Eating keeps my feelings at a tolerable level		.764		.584
Eating helps me to cope with negative thoughts		.857		.735
Eating helps me cope with negative feelings		.840		.706
Eating is my best way of coping with unwanted feelings		.791		.626
Bingeing is something that I can have for myself			.734	.539
I deserve to have a pleasure like binge eating			.891	.794
It’s okay to have the nice experience of binge eating			.826	.682
Bingeing allows me to have something nice for myself			.825	.681
It won’t make a difference if I eat more			.463	.214
I like to binge			.671	.450

Table 5 summarises the fit statistics of the original two-factor EBQ [22], the revised three-factor 25-item EBQ and the short-form three-factor EBQ-18. The result of an incremental model fit test suggests that the final three-factor (18-items) model (EBQ-18) provides a significantly superior fit than the 25-item three-factor model, Δχ^2^ = 393.358, *p <* .001. There is no difference between of the χ^2^ of the Original EBQ (two-factor, 16-items) and the EBQ-18 (three-factor, 18-items), however the EBQ-18 (three-factor, 18-items) demonstrated superior fit across the indicators of goodness-of-fit (χ^2^/df, GFI, IFI, TLo, CFI and RMSEA) compared to the Original EBQ (two-factor, 16-items), as well as compared to the Revised EBQ (three-factor 25-items).

**Table 5:** Summary of fit statistics for three different versions of the eating beliefs questionnaire

Model	χ^2^	df	χ^2^ /df	GFI	IFI	TLo	CFI	RMSEA
1. Original EBQ Two-Factor *(16 items)*	275.384	103	2.674	0.93	0.96	0.95	0.96	.062 (.053–.070)
2. Revised EBQ Three-Factor *(25 items)*	666.268	272	2.450	0.89	0.95	0.94	0.95	.057 (.052–.063)
3. EBQ-18 Three-Factor *(18 items)*	272.91	132	2.038	0.94	0.97	0.97	0.97	.049 (.041–.057)

#### Psychometric properties

The psychometric properties of the measure, and its three subscales, were assessed based on the short-form 18-item EBQ examined in the CFA above.

Table 6 summarises the mean total scores and subscale scores for the total sample and for subgroups of the participants who self-reported to be engaging in a clinical level of binge eating (at least four OBEs per month, with a self-reported average of 10 OBEs per month; BE, *n =* 169) or participants who reported that they did not engage in any OBEs over the previous month (Non-BE, *n* = 481) as reported on the EDE-Q. Results from one-way ANOVAs found significant differences between BE and Non-BE scores on the EBQ subscales with moderate-to-large effect sizes (η_p_^2^): EBQ-18 Total Score: *F(1,648)* = 324.42, *p < .01,* η_p_^2^ *=* .33*;* Negative Beliefs Score^1^: *F(1,228.29)* = 314.67, *p < .01,* η_p_^2^ *=* .40*;* Positive Beliefs Score^1^: *F(1,272.41)* = 186.04, *p < .01,* η_p_^2^ *=* .24, Permissive Beliefs Score: *F(1,648)* = 50.95, *p < .01,* η_p_^2^ *=* .07.

**Table 6:** EBQ-18 subscale group performance

	Total EBQ-18 mean (SD)	Negative beliefs mean (SD)	Positive beliefs mean (SD)	Permissive beliefs mean (SD)
All Participants (*N* = 883)	38.25 (12.87)	12.35 (5.09)	13.68 (5.87)	12.23 (4.93)
Subgroups:				
Non-BE (*n* = 481)	33.20 (10.59)	10.19 (3.65)	11.67 (5.12)	11.33 (4.81)
BE (*n* = 169)	50.68 (11.60)	17.05 (5.48)	17.78 (5.73)	14.40 (4.97)

#### Internal consistency

Cronbach’s alphas (α) were calculated with the full sample (*N* = 883). The revised brief scale (EBQ-18) demonstrated excellent internal consistency, with α = .92 for the EBQ-18 Total Score, α = .88 for the Negative Beliefs Scale, α = .92 for the Positive Beliefs Scale, and α = .88 for the Permissive Beliefs Scale.

#### Criterion validity

The criterion validity of the EBQ-18 was assessed by examining correlations between the EBQ-18 and the two previous versions of the EBQ, as well as examining the correlation with a similar measure, the EDTQ [23]. The EBQ-18 and the original two-factor EBQ [22] were very strongly correlated, *r* = .92, *p <* .01. The EBQ-18 and the 25-item version of the three-factor EBQ identified in the EFA of this manuscript were also very strongly correlated, *r* = .98, *p <* .01. The EBQ-18 and the EDTQ demonstrated a moderate-to-strong positive correlation, *r* = .64, *p <* .01.

#### Construct validity

Convergent validity was assessed by examining the correlations between EBQ subscale scores and the included measures of specific eating disorder symptoms and related psychopathology. The EBQ-18 Total Score, Negative Beliefs Scale and Positive Beliefs Scale correlated significantly and in a positive direction for all the included measures, with one exception - EDCBQ High Standards for Self scale. The Permissive Beliefs Scale correlated significantly and in a positive direction for all the included measures, with three exceptions – BMI, EDE-Q Restraint, and EDCBQ High Standards for Self scale, see Table 7.

**Table 7:** Correlations between EBQ-18 scores and other included measures

	EBQ-18 total score	Negative beliefs scale	Positive beliefs scale	Permissive beliefs scale
Total sample (*N* = 883)				
BMI	.100^a^	.141^a^	.093^a^	.005
DASS-21 Depression	.336^a^	.389^a^	.303^a^	.116^a^
DASS-21 Anxiety	.378^a^	.380^a^	.351^a^	.177^a^
DASS-21 Stress	.350^a^	.391^a^	.352^a^	.092^a^
EDE-Q - Objective Binge Episodes (OBE)	.492^a^	.545^a^	.405^a^	.241^a^
EDE-Q Global Score	.436^a^	.574^a^	.387^a^	.084^b^
EDE-Q Restraint	.153^a^	.297^a^	.122^a^	−.053
EDE-Q Eating Concern	.519^*a^	.626^a^	.465^a^	.157^a^
EDE-Q Shape Concern	.442^a^	.566^a^	.392^a^	.103^a^
EDE-Q Weight Concern	.436^a^	.553^a^	.397^a^	.095^a^
University sample only (*n* = 767)				
EDCBQ Self-Loathing	.401^a^	.429^a^	.324^a^	.213^a^
EDCBQ Demanding/Needing Help	.389^a^	.380^a^	.346^a^	.206^a^
EDCBQ Unassertive/Inhibited	.332^a^	.290^a^	.296^a^	.206^a^
EDCBQ High Standards for Self	.042	.022	.064	.007
EDCBQ Abandoned/Isolated	.355^a^	.358^a^	.363^a^	.120^a^
DERS Total Score	.491^a^	.491^a^	.455^a^	.226^a^
DEBQ Emotional Eating	.676^a^	.609^a^	.673^a^	.323^a^
DEBQ External Eating	.458^a^	.391^a^	.379^a^	.334^a^
EDTQ Total Score	.634^a^	.676^a^	.564^a^	.277^a^
EDTQ – Negative Thoughts	.469^a^	.603^a^	.407^a^	.113^a^
EDTQ – Positive Thoughts	.621^a^	.590^a^	.597^a^	.293^a^
EDTQ – Permissive Thoughts	.557^a^	.482^a^	.474^a^	.383^a^

^a^ = Pearson Correlation is significant at the 0.01 level (two-tailed)

^b^ = Pearson Correlation is significant at the 0.05 level (two-tailed)

## Discussion

The present study sought to develop and assess the validity of a third subscale measuring permissive beliefs about eating to further develop the existing two-factor EBQ in keeping with the cognitive models of binge eating. This study had four main aims: (1) to develop a third subscale for a revised version of the EBQ that aims to measure permissive beliefs about eating; (2) to investigate the factor structure of the revised three-factor EBQ; (3) to present a short-form of the three-factor EBQ; and (4) to examine the psychometric properties of the revised three-factor EBQ. It was hoped that with the addition of the permissive beliefs scale, the EBQ would offer a more comprehensive self-report tool for the measurement of specific metacognitive beliefs about the process of binge eating that are hypothesized to maintain binge eating. The findings of this study supported our hypotheses that the addition of permissive belief items to the existing positive and negative belief items of the EBQ would result in a three factor solution (positive, negative and permissive beliefs about eating), and that the resulting revised version of the EBQ would demonstrate evidence of good psychometric properties including adequate internal consistency, and convergent validity.

These new permissive items were developed with consultation with the relevant literature and assessed for appropriateness/relevance by 10 experts in the field of eating disorders. The 35 proposed items of the revised EBQ were assessed for their fit to the data and their factor structure was examined in an EFA. Following the removal of ten items with low loadings or cross-loadings, an EFA was conducted on the remaining 25 items. The results of this EFA provided support for our hypothesis of a three-factor solution (positive, negative and permissive beliefs) which explained 63.4% of variance. In addition, significant positive correlations were found between each of the three factors (positive, negative and permissive beliefs). To further assess the factor structure, a CFA was conducted on the three-factor model of the EBQ items. This model included a higher-order factor of ‘Eating Beliefs’. Results of the CFA supported the three-factor solution, with the 25-item version of the EBQ providing an adequate to good fit to the data.

The new three-factor EBQ was further developed by forcing evenly sized subscales of the best six items each, resulting in a short-form version of the three-factor EBQ. A CFA was conducted on this model and the three-factor solution for this brief 18-item version of the measure demonstrated good fit to the data. In addition, individual item loadings were all significant, the loadings of the three sub-factors to the higher-order factor were significant, and significant positive correlations were found between each of the three factors (positive, negative and permissive beliefs). The short-form version of the EBQ, the EBQ-18, was found to provide a significantly better fit to the data compared to the 25-item version, and the EBQ-18 demonstrated better results on the goodness-of-fit indices than the original two-factor EBQ [33]. A copy of the resultant three-factor version of the EBQ − 18 can be found in the Additional file 1 of this article.

### Results EBQ-18 psychometric properties

The EBQ-18 was found to be a valid and reliable measure with strong evidence for adequacy on a number of psychometric properties [40]. The EBQ-18 total score and the three subscale scores demonstrated excellent internal consistency with all Cronbach’s alphas over .88. The EBQ-18 demonstrated criterion validity with very strong correlations with previous versions of the EBQ. The EBQ-18 also demonstrated a moderate-to-strong correlation with the EDTQ, this indicates that while the two measures are closely related, measuring a similar construct, but that they are not measuring the exact same construct. The subscale correlations between the EBQ and EDTQ with all corresponding subscales correlating most strongly (e.g. EDTQ negative thoughts subscale correlates more strongly with EBQ negative beliefs scale than the EBQ positive or permissive beliefs scales), however it is important to note that these correlations are small-to-moderate in size and again indicated that though the EBQ-18 and the EDTQ are measuring similar and related constructs, they are assessing different constructs [40]. In addition, the EBQ-18 demonstrated good convergent validity with other measures of related eating disorder symptoms and psychopathology. These significant positive correlations provide evidence of the EBQ-18 convergent validity with measures of related concepts.

However, the relationship between some of the included measures and the EBQ-18 were not straight-forward, particularly in relation to the newest subscale of the EBQ, the permissive beliefs subscale. For example, we observed significant positive correlations between BMI and EBQ-18 total scores, negative beliefs subscale scores, and positive beliefs subscale scores but no relationship was identified between the permissive beliefs subscale and participants’ BMI. This result is interesting as one might expect endorsement of items related to engagement in binge eating episodes would be related to higher BMIs given the known relationship between binge eating and obesity [9]. However, it is also important to consider that binge eating is a behaviour that occurs in individuals at any weight and that the sample used in this study is a general sample with an average BMI in the normal range and that these two factors might be influencing the relationship between BMI and EBQ-18 scores. A similar pattern of correlations was also observed between the EBQ scores and the EDE-Q restraint subscale; significant positive correlations between EDE-Q restraint subscale and EBQ-18 Total score, negative beliefs subscale and positive beliefs subscale. However, no relationship identified between permissive beliefs subscale and EDE-Q restraint subscale. The EDE-Q restraint subscale measures the respondent’s engagement in restrictive eating practices such as dieting and fasting [26]. The lack of a relationship between permissive thoughts and BMI and permissive thoughts and restraint replicate patterns observed in previous studies [23]. Considering that binge eating is associated with restrictive practices in some cases (BN and AN-BP) and not associated with restrictive practices in others (BED) [1], it is not surprising that overall permissive beliefs (e.g. “it’s okay to have the nice experience of bingeing”) are not related to the restraint subscale (e.g., “Have you gone for long periods of time (8 waking hours or more) without eating anything at all in order to influence your shape or weight?”). It would be interesting to investigate this finding further by comparing the correlations between EBQ scores and restraint between different eating disorder subgroups (e.g., BED compared to AN-BP).

These results indicate that the permissive scale is different to the positive and negative beliefs subscales; that it is measuring something about binge eating that is not being assessed by the other subscales. As a result, these discrepancies indicate that the permissive beliefs subscale provides a unique contribution to the EBQ. We believe that this unique contribution makes the EBQ a more comprehensive measure of beliefs related to binge eating. Further, the results of this study have provided more insight into the nature of permissive beliefs, and how they differ from positive and negative beliefs about eating. However, further research is required to better understand the role of permissive beliefs in the maintenance of binge eating.

Importantly, this study provided evidence for the clinical utility of a revised and brief measure; there was a significant difference between the EBQ-18 scores of participants who self-reported to engage in binge eating (4 or more OBEs per month) and participants who reported that they did not engage in binge eating (0 OBEs per month). The clinical utility of the original positive and negative beliefs subscales of the EBQ have been demonstrated in previous studies, across both non-clinical and clinical samples. However, this is the first time that the clinical utility of the permissive beliefs scale has been assessed, and the first investigation of the shorter version of the positive beliefs scale. These findings indicate that the EBQ-18 will provide a useful tool to examine maintaining beliefs in individuals who are seeking treatment for binge eating. However, this examination is only preliminary and further assessment is required within samples of participants with confirmed diagnoses of eating disorders.

### Limitations and future directions

The design of this study was exploratory, and there are certain limitations, warranting further research. First, while we included a large sample, it was nonetheless non-clinical and consisted of a university sample and a community sample. Second, binge eating status was determined by self-report and not verified by an assessment from a mental health professional. Third, the preliminary examination of the psychometric properties of the EBQ-18 presented in this paper provides only a small amount of information regarding the validity, reliability and clinical utility of this measure. We advise that future studies further examine the validity, reliability and clinical utility of the EBQ-18 using both clinical and non-clinical samples. Specifically, other methods of assessing the psychometric properties could be employed by future studies such as applying an estimation method (e.g. WLSMV estimator in Mplus), conducting regression analyses, and assessing whether the factor structure of the EBQ-18 differs depending on factors such as gender or binge eating status. Future studies should also examine the differences in scores between clinical eating disorder groups and non-clinical controls, with binge eating status verified by a clinician interview, such as the EDE. Furthermore, it is important for future studies to examine the EBQ-18’s sensitivity to treatment and test-retest reliability (recent research has examined the utility of the EBQ-18 in both general and clinical eating disorder samples [44]).

### Significance

Permissive metacognitive beliefs are hypothesised to play an important role in the maintenance of binge eating for individuals with eating disorders [19]. The nature and function of the permissive metacognitions assessed by the items in the EBQ-18 were found to be distinct from that of the positive and negative beliefs items. While positive beliefs refer to the role of eating for emotional regulation, and negative beliefs refer to the sense of loss of control over eating experienced by individuals who binge eat, permissive beliefs relate to the notion of binge eating not only as a coping mechanism, but as a pleasant event which may be planned or looked forward to. With the addition of a third scale to measure permissive beliefs, the EBQ-18 offers a more comprehensive tool for measuring the beliefs thought to maintain binge eating in BN and BED. We believe that the revised EBQ-18 will provide a valuable tool for both clinicians and researchers who wish to measure key maintaining cognitions, and examine how these beliefs shift over the course of intervention, in individuals who binge eat.

## Conclusions

This paper presents the EBQ-18, a short-form self-report measure that assesses three types of metacognitions about food and eating thought to be relevant to the maintenance of binge eating behaviour [19]. This new scale demonstrated good internal consistency, as well as good convergent validity self-report measures of eating disorder symptoms, emotional regulation, mood and anxiety. With the addition of a third scale to measure permissive beliefs, the EBQ-18 offers a comprehensive tool to assess the particular beliefs about binge eating that can be addressed in therapy. It is hoped that the revised EBQ will provide a valuable tool for both clinicians and researchers wishing to measure key maintaining cognitions, and how these shift over the course of intervention, in individuals who binge eat.

## Additional file

Additional file 1: EBQ-18 – Eating Beliefs Questionnaire. (DOCX 33 kb)

## Abbreviations

AN: Anorexia Nervosa
AN-BP: Anorexia Nervosa (binge/purge subtype)
ANOVA: Analysis of Variance
BE: Binge eating
BED: Binge Eating Disorder
BMI: Body Mass Index
BN: Bulimia Nervosa
CBT: Cognitive Behavioural Therapy
CBT-E: Enhanced Cognitive Behavioural Therapy
CFA: Confirmatory Factor Analysis
CFI: Comparative Fit Index
CI: Confidence Intervals
DASS-21: Depression Anxiety Stress Scales
DEBQ: Dutch Eating Behaviour Questionnaire
DERS: Difficulty in Emotion Regulation Scale
df: Degrees of freedom
EBQ: Eating Beliefs Questionnaire
ED-CBQ: Eating Disorders Core Beliefs Questionnaire
EDE: Eating Disorders Examination
EDE-Q: Eating Disorders Examination Questionnaire
EDTQ: Eating Disorders Thoughts Questionnaire
EFA: Exploratory Factor Analysis
GFI: Goodness of Fit Index
IFI: Incremental Fit Index
OBE: Objective Binge Eating Episodes
PCA: Principal Components Analysis
RMSEA: Root Mean Square Error of Approximation
SD: Standard Deviation
SPSS: Statistical Package for Social Sciences
TLo: Tucker-Lewis Index

## Footnotes

1 Unequal variances were observed for the responses between BE and non-BE groups for the Negative (Levene’s Test of Equality of Error Variances: *F(1,648)* = 36.915, *p < .01*) and Positive Beliefs (Levene’s Test of Equality of Error Variances: *F(1,648)* = 5.068, *p = .025*) subscales. As the assumptions of homogeneity of variance were not met for two of the subscales, the Welch Robust Tests of Equality of Means have been used.
